# Deep learning detection of retinal detachment: Optical coherence tomography staging and estimation of duration of macular detachment

**DOI:** 10.1371/journal.pone.0329951

**Published:** 2025-09-02

**Authors:** Ansgar Beuse, Inês V. Lopes, Martin S. Spitzer, Vasyl Druchkiv, Carsten Grohmann, Christos Skevas

**Affiliations:** Department of Ophthalmology, University Medical Center Hamburg-Eppendorf, Hamburg, Germany; Saitama Medical Center: Saitama Ika Daigaku Sogo Iryo Center, JAPAN

## Abstract

**Objective:**

To test the applicability of deep learning models for detecting and staging rhegmatogenous retinal detachment (RRD) based on morphological features using two- and three-dimensional optical coherence tomography (OCT) scans.

**Design:**

Retrospective study using deep learning-based image classification analysis of 2D and 3D OCT scans combined with clinical baseline data.

**Subjects:**

Adult patients presenting to the University Medical Center Hamburg-Eppendorf in Germany.

**Methods:**

A total of 252 eyes with RRD and 770 control eyes were included. All OCT scans and clinical baseline data were reviewed and graded. Binary and multiclass classification approaches were applied.

**Main outcome measures:**

Area under the curve (AUC) and precision-recall area under the curve (PR AUC) for detection, stage classification and duration estimation of RRD.

**Results:**

We employed both statistical and deep learning-based approaches using 2D and 3D OCT data. We evaluated an automated 3D OCT classification model in a multiclass analysis to distinguish RRD scans by macula status from a non-RRD group with macula-on cases (PR AUC = 0.66 ± 0.12, AUC = 0.96 ± 0.01) vs. macula-off cases (PR AUC = 0.86 ± 0.07, 0.98 ± 0.01) against non-RRD cases (PR AUC = 1.00, AUC = 1.00) Furthermore, the 3D model was able to classify the duration of macula-off status (< 3 days) with a PR AUC of 0.68 ± 0.2 and a AUC of 0.97 ± 0.2 when compared to a mixed group including longer macular-off, macular-on and non RRD cases. Lastly, manually graded RRD Stages were correlated with best corrected visual acuity (BCVA), as well as macula-off Duration and classified via a 2D model. A 2D model used for RRD stage classification achieved its best performance for stage 4, with a PR AUC of 0.56 ± 0.11 and an AUC of 0.94 ± 0.02.

**Conclusion:**

The machine learning models demonstrated strong performance in classifying RRD stages, macula status and duration based on OCT imaging. These findings highlight the potential of deep learning methods to support clinical decision-making and surgical planning in RRD management.

## Introduction

Rhegmatogenous retinal detachment (RRD) is a serious and potentially sight-threatening ophthalmological emergency. The visual prognosis of RRD depends on multiple factors, most notably the macular involvement at presentation (macula-on vs. macula-off), the duration of central vision loss and the timing of surgical intervention [1–3]. It is well established that patients with an attached fovea have a more favorable visual prognosis, typically achieving a higher visual acuity than those with foveal involvement, regardless of symptom duration [1].

If left undiagnosed or improperly managed, an RRD can progress to foveal detachment, turning it into a time-critical surgical emergency. The acceptable time until surgical intervention in macula-off RRD remains controversial [2]. While earlier studies suggested that macular detachment leads to irreversible functional damage, potentially reducing the urgency compared to macula-on RRD [4], more recent studies demonstrated that photoreceptor cell death begins within the first 12 hours after the detachment and peaks after 2–3 days. Although the underlying cellular and molecular mechanisms are not yet fully understood, these findings suggest that even macula-off cases may benefit from a prompt surgical intervention [3,5]. A recent meta-analysis further supports this, showing that patients treated within three days achieved better visual outcomes than those repaired between days 4 and 7 [6].

Although the optimal timing for surgery in cases of fovea-involving RRD remains undefined, the duration of the foveal detachment is increasingly recognized as a key factor in determining the urgency of surgical intervention. However, accurately assessing the duration of central vision loss can, for several reasons, be challenging in clinical practice [7,8].

Optical coherence tomography (OCT), routinely performed in cases of RRD at the time of presentation, may provide valuable information that support clinicians in estimating the duration of foveal attachment. Martins Melo et al. proposed an innovative staging system based on the sequential outer retinal changes observed on swept-source OCT (SS-OCT) of patients with RRD [8]. The authors identified six distinct stages, which were further supported by observations from prior histological studies.

The use of machine learning algorithms (MLAs) in ophthalmic imaging has been extensively explored across a broad spectrum of conditions, ranging from common ones like diabetic retinopathy and age-related macular degeneration to rarer pathologies such as central retinal artery or vein occlusion [9–12]. In the context of retinal detachment, initial research has demonstrated the feasibility of MLAs, particularly using fundus photography and ultra-widefield imaging [13,14].

## Methods

### Data

This single-center retrospective study included 252 eyes from consecutive patients diagnosed with RRD, as well as 770 eyes without relevant pathomorphological changes, who presented to the University Medical Center Hamburg-Eppendorf, Germany, between January 2018 and January 2022. Only primary RRD cases were included. Exclusion criteria comprised any previous retinal defects or detachment, laser therapy or other prior retinal interventions, regardless of the time of occurrence. After preprocessing, 251 RRD scans were included in the MLA analysis.

The study was approved by the local ethics committee (Ethics Committee Hamburg, ID: 101389-BO) and conducted in accordance with the Declaration of Helsinki. All patient data were anonymized prior to analysis and no access to identifiable information was granted after data collection. The complete dataset was accessed and locked for analysis on February 23, 2025.

A retrospective, anonymized dataset from the University Medical Center Hamburg-Eppendorf (Hamburg, Germany) was used for model training, validation and testing. To ensure balanced representation across all categories, the dataset was organized using stratified 5-fold cross-validation based on RRD stages and the non-RRD control group. This allowed for robust evaluation of stage classification performance, while also enabling independent assessment of macular involvement and detachment duration.

The staging system was based on anatomical changes in the outer retina and the photoreceptor layer, following the six-stage classification proposed by Martins Melo et al (8). The stages were defined as follows: Stage 1: Separation of the neurosensory retina from the retinal pigment epithelium (RPE); Stage 2: Thickening of the photoreceptor layer; Stage 3: Formation of outer retinal corrugations (ORC), further divided in Stage 3a: low frequency ORCs and Stage 3b: high frequency ORCs; Stage 4: Progressive loss of ORC definition with concurrent thickening of the photoreceptor layer; Stage 5: Partly (“moth-eaten”) or complete loss of photoreceptors. For the purpose of MLA analysis s stages 4 and 5 were combined.

OCT-Scans obtained using our TOPCON device were carefully assessed by a senior retina specialist. For quality control, we defined an image quality level (IQV) of 60 as the minimum threshold. Scans with an IQV below 60 were excluded, as lower-quality images did not allow a reliable evaluation of the subtle morphological changes necessary to accurately distinguish between RRD stages. Clinical baseline characteristics and an estimation of the onset of foveal detachment, based on patient history as documented in the electronic medical records, were assessed.

The OCT dataset comprised a diverse patient population of both female and male subjects, with ages ranging from 18 to 93 years. Three-dimensional (3D) volume OCT scans, used for analysis of macular status and detachment duration, and corresponding two-dimensional (2D) B-scans, used for stage analysis, were collected using a Spectral-Domain OCT device (Triton OCT, Topcon Corporation, Tokyo, Japan) during clinical visits. Baseline characteristics of both the RRD and non-RRD control groups are summarized in Table 1.

**Table 1:** Baseline characteristics of RRD group.

**Age**	
Range	19 - 93
Median (SD)	63.90 (13.37)
Median (Q1, Q3)	64. (58., 72.)
**Affected eye**	
Right	138 (54.8%)
Left	114 (45.2%)
**Sex**	
Male	168 (66.7%)
Female	84 (33.3%)
**OCT Staging**	
1	41 (16.3%)
2	39 (15.5%)
3a	50 (19.8%)
3b	95 (37.7%)
4	19 (7.5%)
5	8 (3.2%)
**Macula Status**	
on	63 (25%)
off	189 (75%)
**BCVA first**	
Range	0.00 - 2.70
Mean	1.19 (0.87)
Median (Q1, Q3)	1.00 (0.40, 2.30)
**BCVA last**	
Range	0.00 - 2.30
Mean (SD)	0.61 (0.48)
Median (Q1, Q3)	0.49 (0.28, 1.00)

SD = Standard Deviation. BCVA = best corrected visual acuity.

The study included 252 OCT scans from patients with RRD, with a median age of 64 years, predominantly male (66.7%) and a slight majority involving the right eye (54.8%). Most RRD cases were in stages 3b (38%) and 3a (20%). The macula was detached in 75% of cases. The reported onset of central vision loss was used as a surrogate for the duration of foveal detachment. Inclusion criteria required the absence of prior ocular surgery. Only high-quality OCT scans were included, manually selected and then reviewed and pre-classified by an experienced retinal specialist. For quality control, we defined an image quality level (IQV) of 60 as the minimum threshold. The non-RRD control group consisted of 770 OCT scans from eyes without RRD or other relevant pathomorphological changes. For this purpose, our institutional database was screened for patients over 18 years of age with OCT scans without known retinal disease. All selected scans were manually reviewed to confirm the absence of retinal detachment, macular pathology or other structural anomalies.

The non-RRD control group was age-matched to the RRD group, with an age range of 18–92 years (median age 49) and a gender distribution of 47% male and 54% female.

The OCT scans used in this study were acquired with the Topcon DRI Triton Plus device, using a horizontal 3D wide volume scan mode (12 mm x 9 mm) with a resolution of 512 x 992 pixels. Each scan typically consisted of 512 columns, 992 rows and 256 slices. The fixation point was centered on the fovea; if central fixation was not possible, an external fixation light was used for the fellow eye. The average slice thickness was 0.035 mm, with an axial resolution of 0.0026 mm.

In the 2D deep learning model, all scans were interpolated to 496 x 496 pixels. For the 3D model, the volumetric data were resampled to 256 x 256 x 64 voxels, preserving the relative spatial structure across axes.

### Model and statistics

We used Python (version 3.9.7, Python Software Foundation, Beaverton, USA) to manage the data infrastructure and perform statistical analyses. The deep learning framework for this project was developed using PyTorch, running on a Quadro RTX 8000 GPU (NVIDIA, Santa Clara, CA, USA) [15].

For statistical analysis of baseline characteristics, we used R (Version 4.4.2.). Incidence of macula-off status and the corresponding confidence intervals were calculated for each OCT stage. These incidences were then compared pairwise using Fisher’s exact test, and the p-values were adjusted using the Holm method. The duration of macula-off status and BCVA were summarized using the median and quartiles. Pairwise comparisons across OCT stages were conducted using the Mann-Whitney U test.

We applied transfer learning by utilizing a pre-trained residual network (ResNet) convolutional neural network (CNN) model for both two- and three-dimensional analyses. For 2D OCT slide classification, a ResNet-18 model comprising 18 layers was used, while a 3D ResNet-18 was implemented for volumetric analysis [16,17]. These models employ shortcut connections to mitigate issues such as vanishing gradients and training divergence.

The tested model architecture begins with a convolutional layer followed by max pooling. This is followed by four convolutional blocks, each containing two residual units with two convolutional layers per unit. Our model then uses an averaging layer for pooling with spatial dimension shrinkage to produce a single fully connected layer. Unlike the 2D model, the 3D model incorporates 3D convolutional layers, enabling the automatic capture and processing of volumetric data with further spatial and temporal dependencies.

For optimal configuration, an Adam algorithm (adaptive moment estimation) was employed with a learning rate of 5x5-5 for weight update optimization [18].

Separate PyTorch data loaders were implemented for training, validation and test sets For the multiclass classification, the algorithm was configured to produce up to six output classes. Each approach had a pre-trained ImageNet configuration before being fine-tuned on the study data [19]. CrossEntropyLoss was used as criterion. For the 2D model, an early stopping method was applied. During training and validation, gradients were processed, and model weights were updated and saved accordingly. We excluded updating the gradient for the test or evaluation phase, for which we selected the best weights from previous steps. The test set consisted of data unseen during training or validation. The hyperparameters used in model training are listed in Table 2. All results were bootstrapped, and 95% confidence intervals calculated and reported.

**Table 2:** Hyperparameters of the two- and three-dimensional models.

Hyperparameter	Variable
*Two-Dimensional*	
Shape	496px x 496px
Layers	1,16,1
Criterion	CrossEntropyLoss
Optimizer	Adam
Learning Rate	5x5^-5^
Batch size	20
Epochs	30
Early Stopping Epoch	10
*Three-Dimensional*	
SHAPE	256px x 256px x 64
Layers	1,16,1
Criterion	CrossEntropyLoss
Optimizer	Adam
Learning Rate	5x5^-5^
Batch size	6
Epochs	20

px = pixel.

Multiclass classification comparing non-RRD scans with macula-on and macula-off RRD scans. The PR AUC indicate moderate discriminative performance for macula-on (PR AUC of 0.66) and macula-off (PR AUC of 0.86) cases. ROC analysis shows high classification performance across all groups, with AUC values > 0,95, reflecting high sensitivity and specificity.

### Preprocessing

For our 2D approach, data augmentation was applied using the Albumentations library within the PyTorch framework to enhance training variability [20]. The following transformations were utilized during training: random shifts, scaling, rotation and resizing through interpolation. In contrast, validation and test sets were resized without any further augmentation (20). All processed images were normalized and subsequently converted into PyTorch tensors using a channel-first structure. For the 3D tasks, DICOM volumes were preprocessed as stacked tensors via normalization, and interpolated. The 3D-Approach utilized a trilinear calculation. For the three configurations, an early stopping mechanism was implemented using the macro-averaged one-vs-rest average precision metric. Training was halted when the predefined patient threshold of 10 epochs was reached without improvement. The model checkpoint with the best validation score was retained to prevent overfitting and reduce computational effort. The final fully-connected layer is sized to the task’s label count (2, 3 or 6).

### Cross validation

To ensure robust model evaluation, the dataset was split and stratified by stage to be then randomly divided into five relatively evenly balanced folds. We employed a two-level cross-validation loop. The outer loop treats one of the five folds as an untouched test set, while the inner loop cycles through the remaining four folds each time using one for validation and the other three for training. For each iteration, one-fold was used as the test set, while the remaining four served for training and validation purposes. Each run through has been used independently for the three different calculations.

Some controls allowed usage of multiple eyes. The dataset was distributed into training, validation and test sets, respectively, with an approximate ratio of 80:20. Class imbalance was addressed by maintaining label proportions across all folds using stratified five-fold-cross-validation. Model selection and performance reporting were based on macro-averaged metrics, especially Average Precision and ROC AUC, applied for both the training/validation folds and the independent test hold out set, respectively). The number of cases and split ratios for training, validation and testing are stated in Table 3. For the first iteration a total of 648 scans were used for training, 176 for validation and 197 for testing.

**Table 3:** Example of cases and split ratio regarding training, validation and testing sets.

	Labels						
	Non-RRD	1	2	3a	3b	4	5
**Training**	498	25	23	29	57	11	5
**Validation**	126	8	8	10	19	4	1
**Test**	146	8	8	10	19	4	2

RRD = rhegmatogenous retinal detachment

## Results

### Data characteristics

This single-center, retrospective study included 252 eyes with RRD and a total of 1,021 OCT scans, with 251 RRD scans mobilized for MLA analysis after preprocessing. The RRD cohort ranged in age from 18 to 93 years, with a mean age of 63.41 years. The majority of participants consists of 67% male and 33% were female. The right eye was affected in 55% of cases and the left eye in 45.2%. Macula-off cases accounted for 75% of the cases, while 25% were classified as macula-on. Regarding the OCT staging, the most frequent stages were 3b (38%) and 3a (20%), with fewer cases in stages 4 (7.5%) and 5 (3%). Best-corrected visual acuity (BCVA) at presentation ranged from 0.00 to 2.70 LogMAR (mean: 1.19) and improved postoperatively to 0.00–2.30 LogMAR (mean: 0.61).

### Model performance

**Fig 1:**
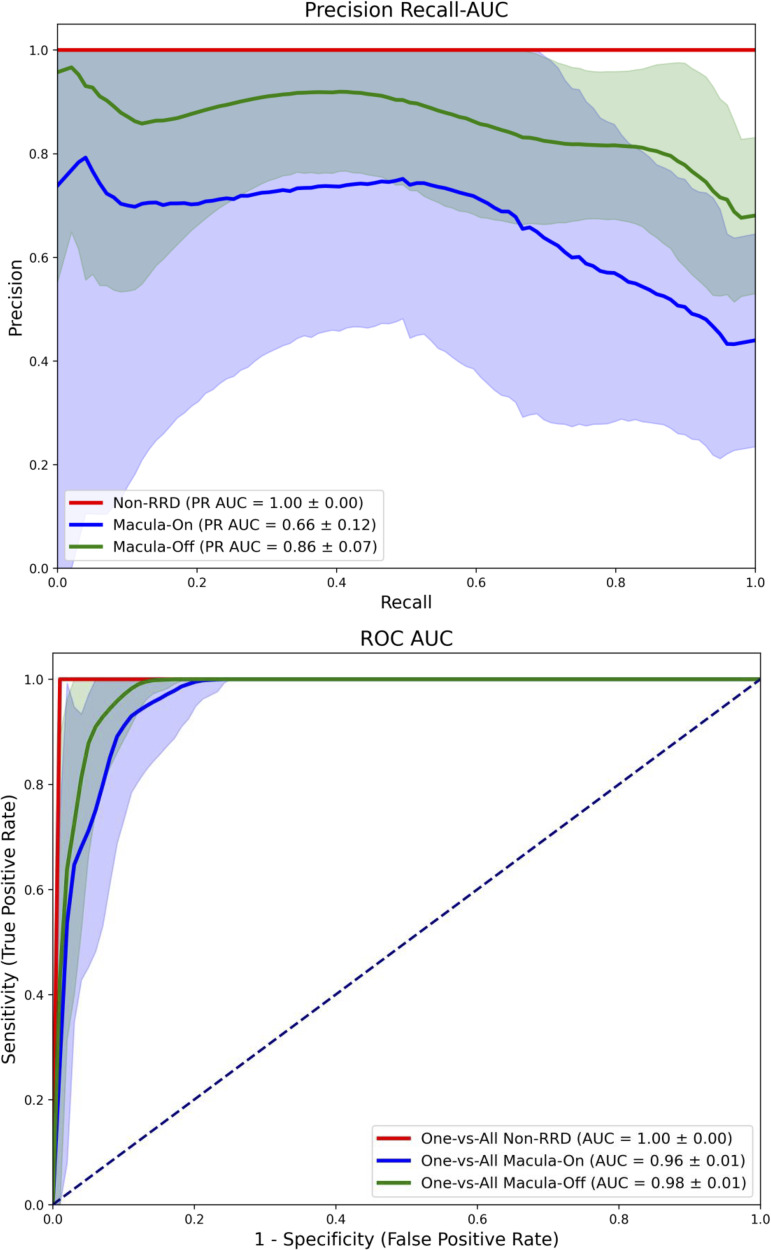
Multiclass classification of non-RRD, macula-on RRD and macula-off RRD scans. PR AUC values of 0.66 for macula-on and 0.86 for macula-off indicate moderate performance with some variability. ROC curves show high classification performance, with AUC values exceeding 0.95 for all classes demonstrating strong sensitivity and specificity.

**Fig 2:**
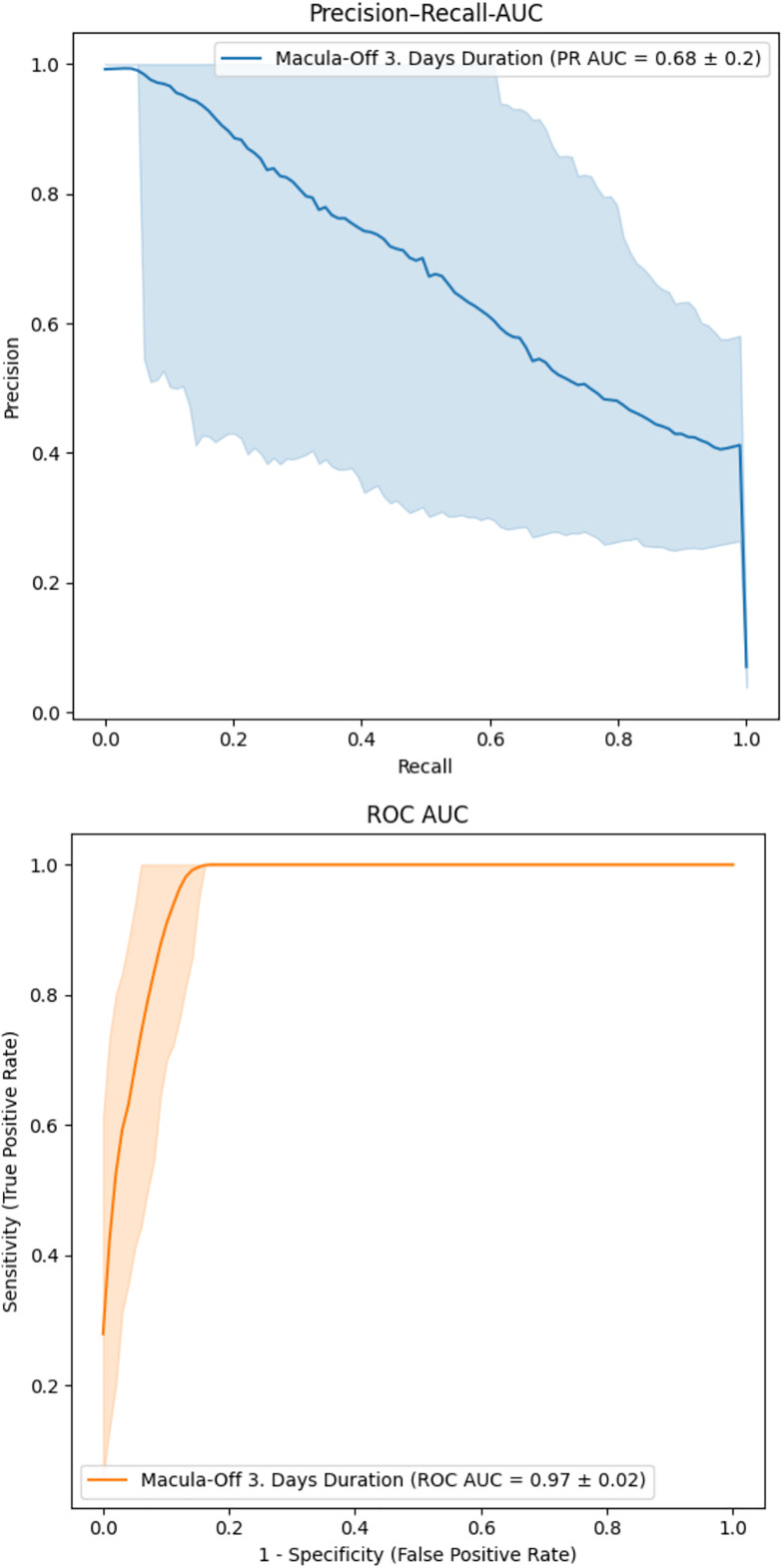
Binary classification distinguishing recent macula-off RRD (≤ 3 days) from other cases (non-RRD, macula-on and longer (> 3 days) macula-off cases). The PR AUC of 0.68 ± 0.2 indicates moderate performance with some variability. The ROC curve shows strong classification performance, with an AUC of 0.97 ± 0.2, reflecting high sensitivity and specificity.

Correct classification using the 3D model to distinguish between macula-on, macula-off and non-RRD cases was achieved with a strong performance metrics. For macula-off cases, the model reached a PR AUC of 0.86 ± 0.07 and an AUC of 0.98 ± 0.01. For macula-on cases, it achieved a PR AUC of 0.66 ± 0.12 and an AUC of 0.96 ± 0.01 for macula-on cases. (Fig 1). In the direct automatic 3D classification of macula-off RRD cases with a symptom duration less than 3 days versus those with a duration of more than 3 days, the model demonstrated a (PR AUC of 0.68 ± 0.2, indicating moderate classification performance with variability across thresholds (Fig 2). The corresponding AUC was 0.97 ± 0.20, reflecting strong overall performance in terms of sensitivity and specificity.

While the PR curve showed reduced precision at lower recall levels, the AUC curve maintained a consistently upward trajectory, suggesting robust model performance across all decision thresholds.

**Fig 3:**
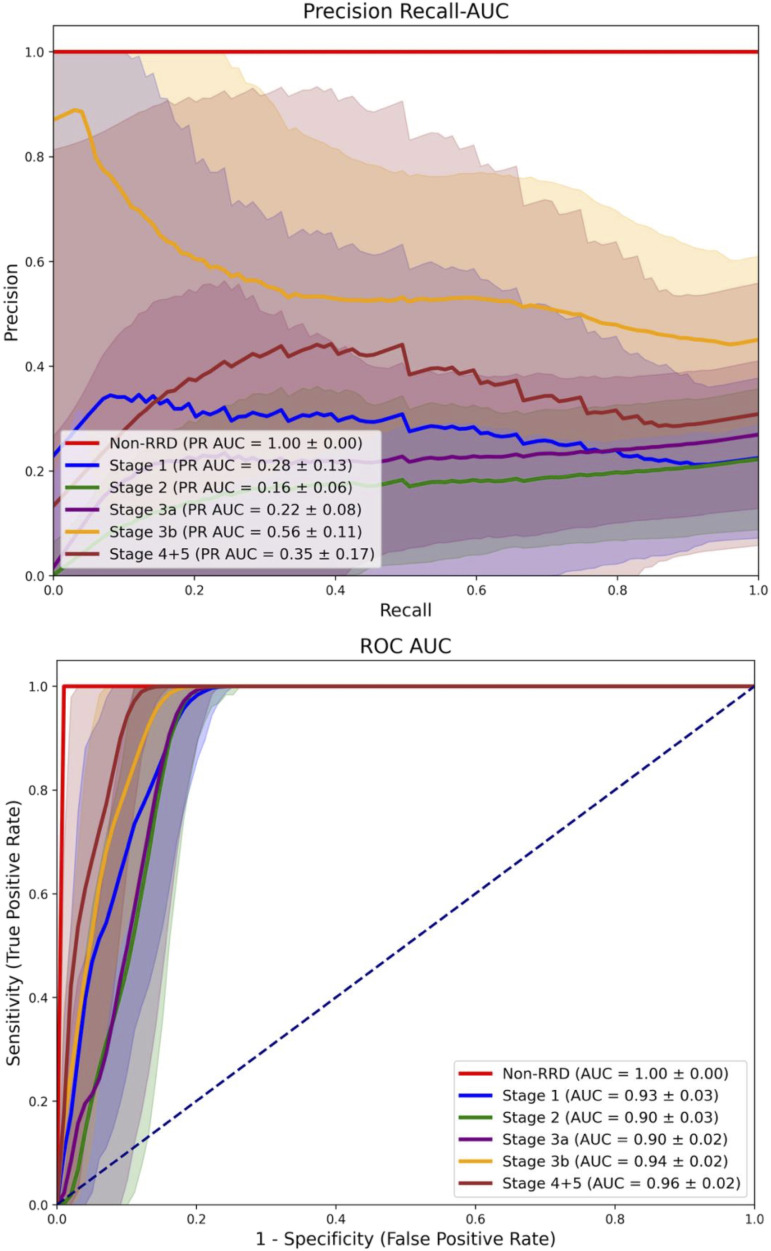
Multiclass classification performance across different RRD stages and non-RRD controls. The models shows variability in performance across the six classes. The non-RRD class achieved the highest PR AUC (1.00), while Stage 2 showed the lowest performance (0.56 ± 0.11). The highest PR AUC among the RRD stages was observed for stage 3b (0.56 ± 0.11). AUC values ranged from 0.90 to 1.00 across all classes, reflecting overall high sensitivity and specificity.

For the 2D RRD stage classification the AUC illustrate classification performance across six classes, with AUC values ranging from 0.90 to 1.00 (Fig 3). For non-RRD, the PR AUC and AUC is 1.00, resembling strong classification. Stage 1 has an AUC of 0.93, while for Stage 2, 3a, 3b, and 4 + 5, the AUC values remain high (0.90–0.94). In the early stages, performance is more challenging, with Stage 1 reaching an PR AUC of 0.28 ± 0.13 and  improves slightly with an PR AUC of 0.22 ± 0.08, while Stage 3b shows the highest PR AUC among the RRD stages, achieving 0.56 ± 0.11. Stage 4 + 5 follows with PR AUC 0.35 ± 0.17, reflecting reasonable classification but some overlap with other stages.

### Statistics

The analysis of macula-off duration and BCVA (LogMar) in relation to OCT grading reveals that higher OCT grades are significantly associated with increased macula-off duration. In particular, Stages 4 and 5 exhibit the longest median duration (Figs 5 and 7). Statistical comparisons between OCT stages show significant differences, particularly between intermediate stages (e.g., 3b) and advanced stages (e.g., 4).

**Fig 4:**
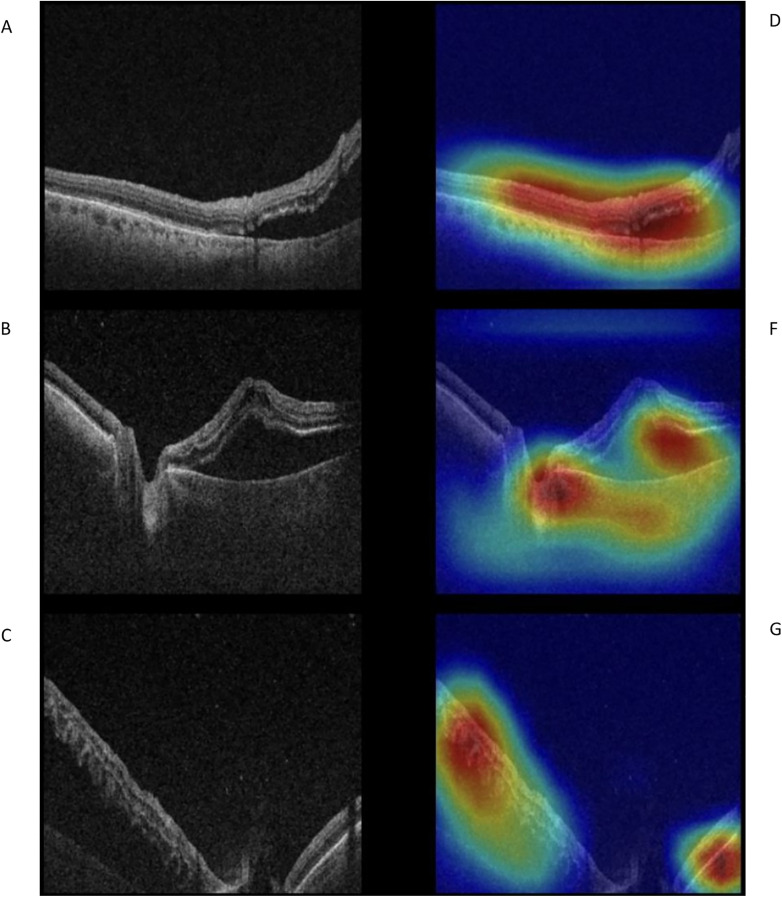
Grad-CAM visualizations highlighting model attention for RRD stage classification. Two-dimensional GRAD-CAM feature map illustrate the estimated regions used by the model to classify RRD stages. On the left side is the true stage depicted and, on the right, the correctly estimated stage. Panels *A-C show original OCT scans labeled as Stage 1, Stage 3 and Stage 4, respectively. Panels D-F display the corresponding correctly predicted scans for the same stages.*

**Fig 5:**
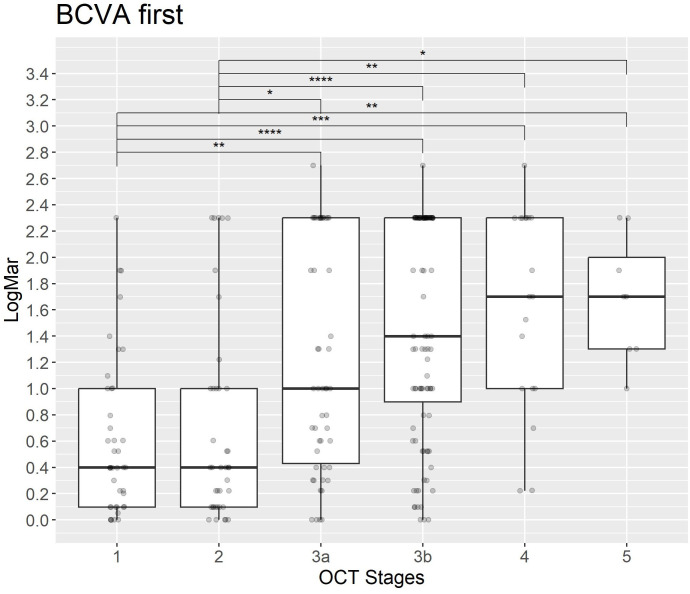
BCVA (LogMAR) at initial presentation across OCT-based RRD stages. The boxplot shows BCVA values, measured in LogMAR, at the first clinical assessment across different manually graded OCT stages of RRD. A trend toward poorer visual acuity (higher LogMAR values) is observed in more advanced stages (3a, 3b, 4, and 5).

**Fig 6:**
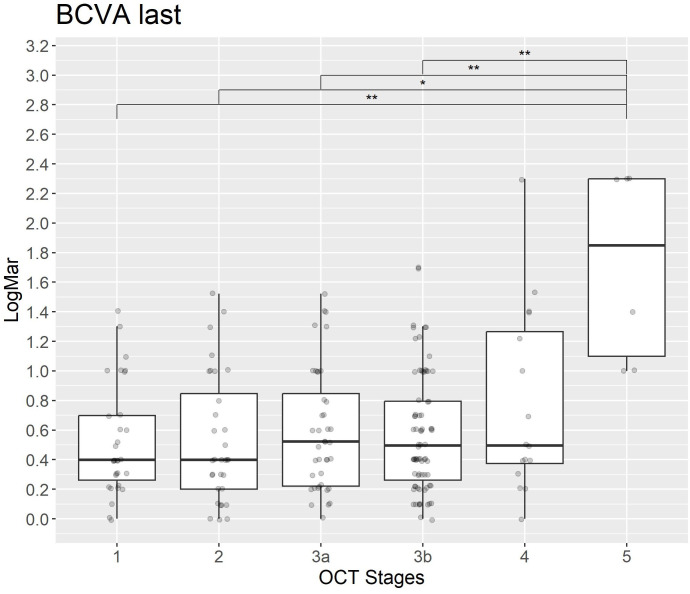
BCVA (LogMAR) at final follow-up across OCT-based RRD stages. The boxplot shows the distribution of BCVA (LogMAR) values, at the time of the last clinical follow-up across the different manually graded OCT stages. Visual outcomes remain worse in higher stages, indicating that initial OCT grading may correlate with long-term visual prognosis.

**Fig 7:**
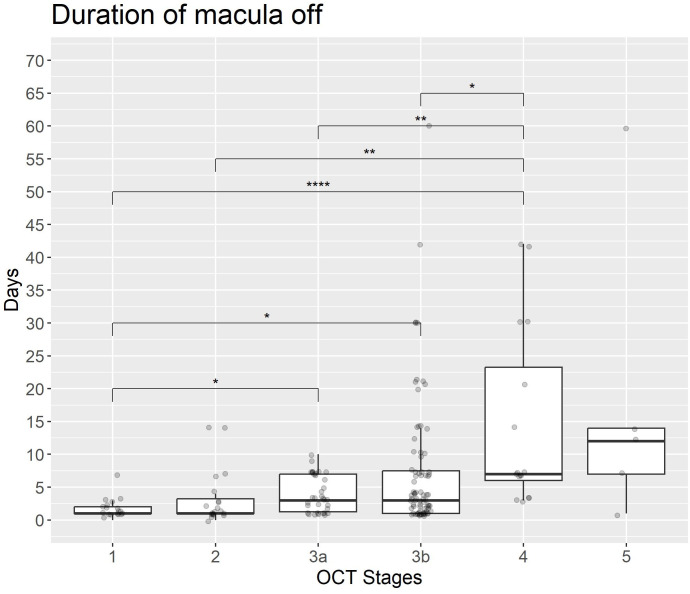
Duration of macula-off status across OCT-based RRD stages. The boxplot illustrates the duration of macular detachment (in days) across different manually graded OCT stages of RRD. A trend toward longer macula-off duration is observed in more advanced stages, particularly Stage 4 and Stage 5.

Similarly, BCVA (LogMar) at the initial assessment worsens with increasing OCT stage. Early stages (1 and 2) present with lower median LogMar values, while advanced stages (4 and 5) show the higher LogMAR values.

This trend persists at the final assessment (Fig 6), where later stages continue to demonstrate significantly worse visual outcomes. These differences were statistically confirmed using Holm-adjusted p-values, highlighting a robust association between OCT-based RRD staging, macula-off duration and visual function at both timepoints.

### Feature map

To enable visual interpretation of the model´s decision-making process, we implemented a 2D GRAD-CAM (Gradient-weighted Class Activation Mapping) module in PyTorch for the OCT stage classification task [21]. For illustration, we randomly selected correctly classified test images from multiple folds and visualized the attention maps after applying the argmax function to the model’s output layer.

## Discussion

We present significant findings that validate previous results regarding the relationship between OCT-based grading, macula-off duration and visual acuity [6,8,22]. Figs 5 and 6 illustrate the distribution of BCVA (LogMAR) at baseline and follow-up, respectively. In both a clear decline in visual acuity is observed with increasing OCT stage. Early-stage detachments (OCT stages 1 and 2) are associated with lower LogMAR values, indicative of better visual function, whereasmore advanced stages (4 and 5) show significantly higher LogMAR values, corresponding to severe visual impairment. These differences are statistically significant after correction using the Holm method, supporting prior findings that higher OCT grades correlate strongly with poorer visual outcomes both at presentation and at follow-up.

Fig 7 further illustrates the relationship between macula-off duration and OCT stage. A progressive increase in duration is evident from early (stages 1 and 2) to intermediate (3a, 3b) and more advanced stages (4,5). While median duration remains relatively low in early OCT stages, a marked increase is observed in later stages, suggesting that rather higher-grade detachments are associated with prolonged macula-off duration. These explorative results emphasize the importance of early diagnosis and intervention to prevent further progression and preserve visual function. They also provide further support for the potential use of OCT-based grading as an objective and data-driven tool for assessing disease chronicity.

Building on these associations, we evaluated the performance of MLA for automated classification of macula-on from macula-off RRDs versus non-RRD cases using a 3D model. The PR AUC for this multi-class task was 0.66 ± 0.12 and 0.86 ± 0.07 for macula-on and macula-off cases respectively, indicating substantial classification performance with some variability. AUC scores further underscored the model´s strong performance, with values of AUC of 0.96 ± 0.01 and 0.98 ± 0.01, for macula-on and macula-off, respectively, highlighting high sensitivity and specificity.

These results demonstrate the model´s robust diagnostic capabilities, while also reflecting areas where trade-offs between precision and recall may affect performance.

To further investigate the potential clinical utility of the approach, we tested the MLAs ability to distinguishing macula-off cases with an estimated duration of less than 3 days from a heterogeneous comparison group consisting of control cases, macula-on cases and macula-off cases older than 3 days. Here, the PR AUC was 0.68 ± 0.2, suggesting moderate discriminative performance with notable variability. However, the AUC reached 0.97 ± 0.02, reflecting high overall sensitivity and specificity. These findings highlight the model’s overall discriminative ability while also reflecting the challenges in precisely distinguishing between these clinical subgroups.

Lastly, we evaluated the model´s ability to directly classify OCT-based RRD stages. In this multi-class task, the PR AUC showed considerable variability across classes. The non-RRD group achieved the highest accuracy (PR AUC of 1.00), demonstrating excellent model reliability for healthy scans. The best PR AUC among the RRD stages is observed in Stage 3b (0.56 ± 0.11), followed by Stages 4 + 5 with 0.35 ± 0.17. The PR AUC reveals substantial challenges in early-stage classification with relatively low PR AUCs highlight limitations in the model’s current discriminative performance. This study represents a retrospective and exploratory analysis, and the findings should therefore be interpreted with caution. Larger datasets are essential to improve classification performance and to enable a more robust evaluation of the model’s clinical applicability.

These findings underscore the potential of MLA’s not only to differentiate RRD cases from normal controls, but also to accurately identify macular involvement and disease onset. Such applications could support clinical decision-making by assisting in diagnosis, enabling triage for surgical intervention and optimizing clinical workflows. In particular, the integration of feature maps not only allows for visualization of possible regions of pathomorphological interest, but also aids in assessing the reliability of model predictions (Fig 4).

This study further identifies robust relationships between OCT-based RRD grading, macula-off duration and visual acuity. Our analysis demonstrates clear and statistically significant differences between OCT stages and their respective clinical implications (Figs 5 and 6). After adjusting for multiple comparisons using the Holm method, we observed a pronounced decline in visual acuity (logMAR) from early (stages 1 and 2) to advanced stages (stages 4 and 5). These results highlight the crucial importance of early detection and timely surgical intervention to preserve visual function.

Additionally, Fig 7 illustrates a progressive increase in macula-off duration with higher OCT grades. This further reinforces the importance of prompt diagnosis and treatment to prevent extended macular detachment and associated visual loss. This data-driven approach supports the clinical importance of OCT-based RRD staging in managing and prognosticating patient outcomes. Integrating feature maps enhances visualization by pinpointing regions of pathomorphological interest. This can help to validate model predictions and provide a deeper understanding of RRD progression.

MLAs have been extensively investigated in Ophthalmology for a range of prevalent conditions, including diabetic retinopathy, glaucoma, retinal venous occlusion, as well as for less common entities such as central retinal artery occlusion [9–12]. In the context of retinal detachment, prior studies have primarily focused on detection using fundus photography or ultra-widefield imaging [13,14]. Our study expands upon this foundation by applying pre-trained MLA’s for classifying macular status in RRD, distinguishing RRD stages and identifying macula-off cases with short symptom duration (Figs 1-3.). The models achieved high sensitivity and specificity, particularly in differentiating between RRD stages and detecting early macular involvement.

These findings highlight the practical potential of MLAs in supporting timely diagnosis, facilitating surgical triage, and streamlining clinical workflows, for ultimately improving patient care. Importantly, in regions with limited access to ophthalmologic care, such as rural or underserved areas, such models could provide valuable pre-screening support. Such tools may help prioritize patients based on disease severity and urgency, ensuring that those in need of prompt surgical intervention are identified and referred more efficiently.

While our findings are promising, several limitations must be addressed. A key limitation of this study is the relatively small and homogeneous sample size. Future research should aim to incorporate a more diverse population across age groups, ethnic backgrounds, and clinical presentations to enhance the generalizability and applicability of the results. Furthermore, the dataset was limited to a single OCT device and central, non-widefield scans, which may have restricted the detection of peripheral RRDs, particularly macula-on cases not centrally located. This highlights a structural limitation of relying solely on standard OCT volume scans.

From a methodological perspective, future studies should consider the integration of data from various OCT devices and other ophthalmologic imaging modalities such as fundus photography or ultra-widefield imaging. Multimodal approaches could improve classification performance and enable a more holistic assessment of retinal detachment. While our current binary and multi-class models provide a foundation, expanding to multi-label classification could allow simultaneous recognition of multiple pathological features, leading to a more nuanced clinical insight. Another limitation lies in the restricted spectrum of RRD etiologies included in this study. Future investigations should encompass a wider range of retinal detachments, including tractional or exudative types, to evaluate MLA applicability across diverse clinical scenarios.

Despite these limitations, our study contributes significantly to the current understanding of MLA use in retinal detachment. First, we validate prior findings showing a strong correlation between OCT-based RRD staging and clinical implications, such as macula-off duration and visual acuity outcomes. Second, we demonstrate that MLAs can not only differentiate between various RRD stages, but also detect macula-off cases with symptom duration under three days. These compatibilities highlight the potential of MLAs to assist in early diagnosis, support triage decisions for surgical intervention and ultimately streamline clinical workflows.

## Abbreviations

2D: two-dimensional
3D: three-dimensional
AUC: area under the curve
BCVA: best corrected visual acuity
CI: confidence interval
MLA: machine learning algorithm
ORC: outer retinal corrugations
PR: precision recall
px: pixel
ResNet: Residual Network
RPE: retinal pigment epithelium
ROC: receiver operating characteristic
RRD: rhegmatogenous retinal detachment
SD: standard deviation
